# A Proposal for an Ultrasensitive Label-Free Optical BioMEMS Platform Based on a DBR-Michelson Interferometer for Cancer Detection and Therapeutic Applications

**DOI:** 10.3390/s26185913

**Published:** 2026-09-18

**Authors:** Bocar Ndiaye, Naima Brahiti, Kazem Nouri, Taha Azad, Kian Jafari

**Affiliations:** 1 Interdisciplinary Institute for Technological Innovation (3IT), Faculty of Engineering, Université de Sherbrooke, Sherbrooke, QC J1K 0A5, Canada; bocar.ndiaye@usherbrooke.ca (B.N.); naima.brahiti@usherbrooke.ca (N.B.); 2 Department of Pathology and Laboratory Medicine, Faculty of Medicine, University of British Columbia, Vancouver, BC V6T 2B5, Canada; kazem.nouri@vch.ca; 3 Department of Microbiology and Infectious Diseases, Faculty of Medicine and Health Sciences and Centre de Recherche du CHUS, Université de Sherbrooke, Sherbrooke, QC J1E 4K8, Canada; taha.azad@usherbrooke.ca; 4 Université de Sherbrooke Cancer Research Institute (IRCUS), Sherbrooke, QC J1E 4K8, Canada; 5 Laboratoire Nanotechnologies Nanosystemes (LN2), Université de Sherbrooke, Sherbrooke, QC J1K 0A5, Canada

**Keywords:** label-free biosensor, Michelson interferometer, MOEMS, optomechanical transduction, parylene-C, signaling pathways, silicon-on-insulator, cancer therapy

## Abstract

Early detection of cancer remains challenging due to the extremely low concentration of biomarkers present during initial disease stages. Dysregulation of key signaling pathways, including cyclin-dependent kinase (CDK) networks, drives uncontrolled cell proliferation and tumor progression, underscoring the need for highly sensitive, label-free biosensing technologies. Conventional analytical methods such as ELISA and PCR offer reliable detection but require complex sample preparation, fluorescent labeling, and long processing times, limiting their suitability for rapid diagnostics. This work introduces a novel label-free Biological Micro-Opto-Electro-Mechanical System (BioMEMS) platform based on an unbalanced Michelson interferometer with DBR mirrors to interrogate the minute spectral shifts induced by biomolecular interactions. A microcantilever is suspended above a silicon-on-insulator (SOI) waveguide, where the specific binding of biomarkers generates compressive surface stress, causing a downward deflection that reduces the cantilever-to-waveguide gap. This displacement modulates the effective refractive index of the sensing arm through enhanced evanescent-field coupling. The resulting phase variation is further detected through the high-sensitivity Michelson interferometric architecture, enabling ultrasensitive spectral interrogation. Numerical simulations using COMSOL Multiphysics demonstrate a narrow full width at half maximum (FWHM) of 27.6 nm, a quality factor (Q) of 53.34, and an overall sensitivity of 75.74 µm/(N/m). These results highlight the potential of the proposed platform for early cancer detection and precise monitoring of dysregulated signaling pathways.

## 1. Introduction

The ability to detect diseases at their earliest stages remains a major challenge in modern healthcare, as diagnostic delays are often associated with irreversible disease progression and reduced treatment effectiveness [1]. In many pathological conditions, including cancers, viral infections, and neurodegenerative disorders, clinical symptoms typically emerge only after substantial biological alterations have already taken place. As a result, late-stage diagnosis significantly compromises therapeutic outcomes while increasing both patient risk and healthcare burden.

Particularly in the context of cancer biology, understanding and regulating these biological alterations is of paramount importance [1]. Cell proliferation is crucial for the growth, development, and maintenance of multicellular organisms [2]. This intricate phenomenon is tightly regulated by the cell cycle, a highly orchestrated sequence of events that governs a cell’s life from birth to division. Numerous pathways, such as the cyclin-dependent kinase (CDK) pathway [3], the Hippo pathway, p53 pathway, Ras-MAPK pathway [4], and retinoblastoma (Rb) pathway [5], are essential for regulating cell proliferation [6]. Aberrations and dysregulation of these regulatory mechanisms underpin the unchecked growth characteristic of malignancies, emphasizing the significance of comprehending and manipulating these intricate molecular processes in research and clinical applications [7].

A key factor underlying this challenge is the extremely low concentration of biomarkers during the early stages of disease development [1]. At these initial phases, the associated biological signals are inherently weak and difficult to distinguish from background noise. This fundamentally stems from three concurrent physical phenomena: thermomechanical noise intrinsic to the sensing structure, non-specific adsorption events that generate spurious signals indistinguishable from true binding, and viscous damping in liquid media that degrades the mechanical quality factor [8,9]. Consequently, reliable early detection requires sensing technologies capable of capturing extremely subtle physical changes induced by biomolecular interactions.

However, despite significant advances in this domain, existing detection techniques still face several limitations in addressing this requirement. Although the identification of target biomarkers relies on specific biological interactions, routine analytical methods such as Enzyme-Linked Immunosorbent Assay (ELISA) or Polymerase Chain Reaction (PCR) remain costly, time-consuming, and require complex fluorescent labeling [10]. Moreover, both conventional and emerging approaches still rely on complex sample preparation and sophisticated instrumentation, while remaining limited in their ability to reliably detect extremely low biomarker concentrations at early disease stages [11]. Recent research has emphasized that integrating Micro-Opto-Electro-Mechanical Systems (MOEMSs) represents a promising alternative strategy for achieving the high-precision displacement sensing required for these applications [12,13,14,15]. Consequently, the development of label-free MOEMS biosensors has become increasingly attractive [16,17,18,19].

Current biosensing platforms often rely on traditional Surface Plasmon Resonance (SPR) sensors or microfluidic devices, which face challenges related to biocompatibility and detection limits. Notably, the use of flexible polymers such as Parylene-C has emerged as a promising solution for improving biocompatibility and structural compliance in sensing applications [20,21]. Although SPR-based biosensors can achieve high sensitivity through refractive index variations at metal–dielectric interfaces, they suffer from intrinsic optical losses and limited integration capability [22,23]. More recently, Chen et al. investigated evanescent-wave-based optical biosensors and demonstrated their strong potential for healthcare applications, although their performance remains limited when detecting extremely low biomarker concentrations [24].

To overcome these limitations, on-chip optical resonators have shown great potential, but their sensitivity is often hindered by background noise [25]. Such noise contributions may originate from thermal fluctuations, optical losses, or environmental perturbations, which can degrade the effective resolution of resonant systems [25,26]. Recent studies confirm that a platform based on a one-dimensional (1D) photonic crystal coupled with a cantilever, creating an adjustable defect width cavity, significantly amplifies light–matter interactions for ultrasensitive detection [27,28]. In this context, Gholami et al. [27] demonstrated that MOEMS platforms based on defect-engineered photonic structures can significantly enhance sensitivity through optical confinement effects. Similarly, Zhang et al. [29] investigated an integrated optomechanical cantilever–waveguide sensor and showed that evanescent-field modulation enables efficient biosensing, although the mechanical response remained limited by the stiffness of silicon-based structures.

**Figure 1:**
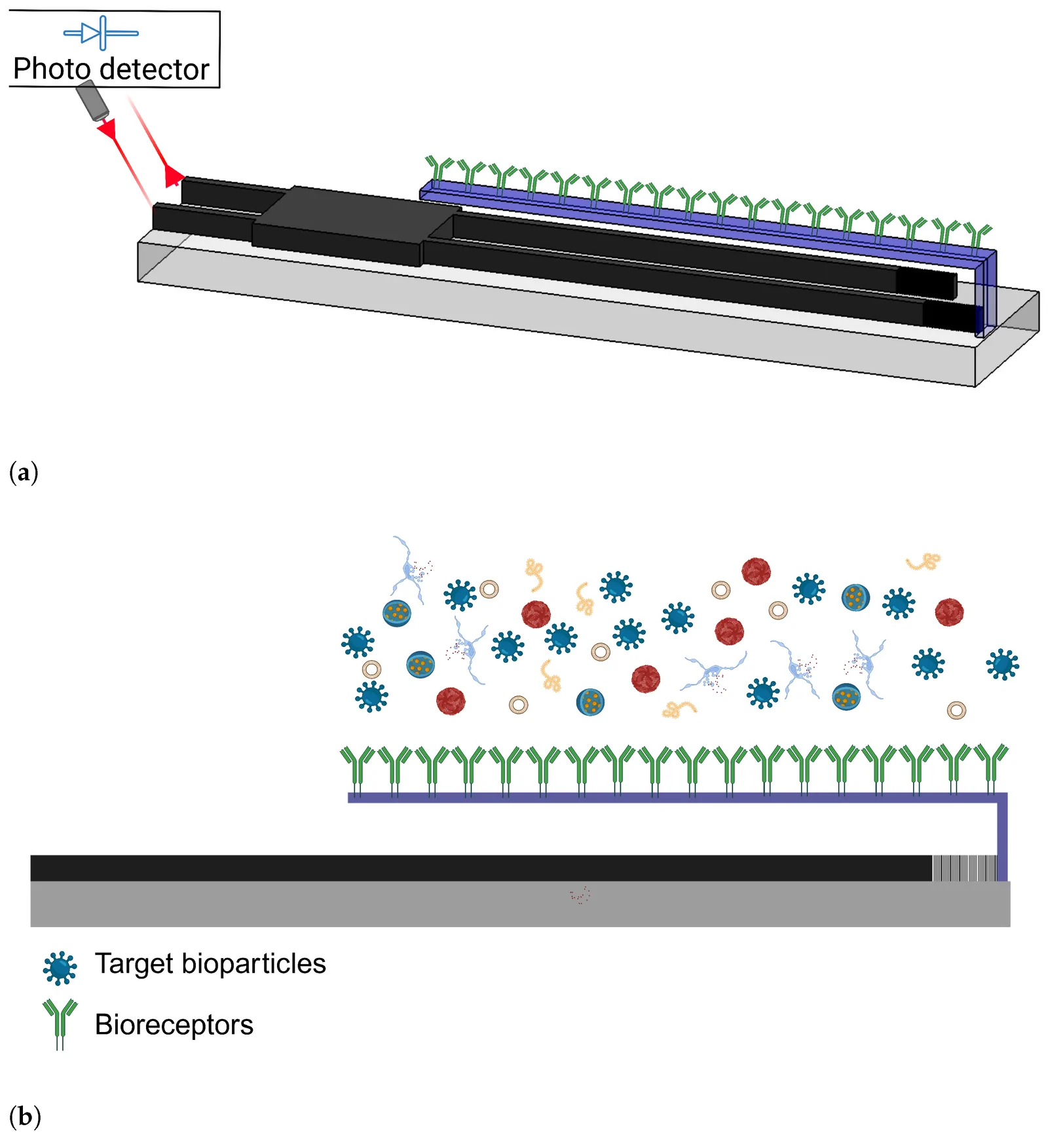

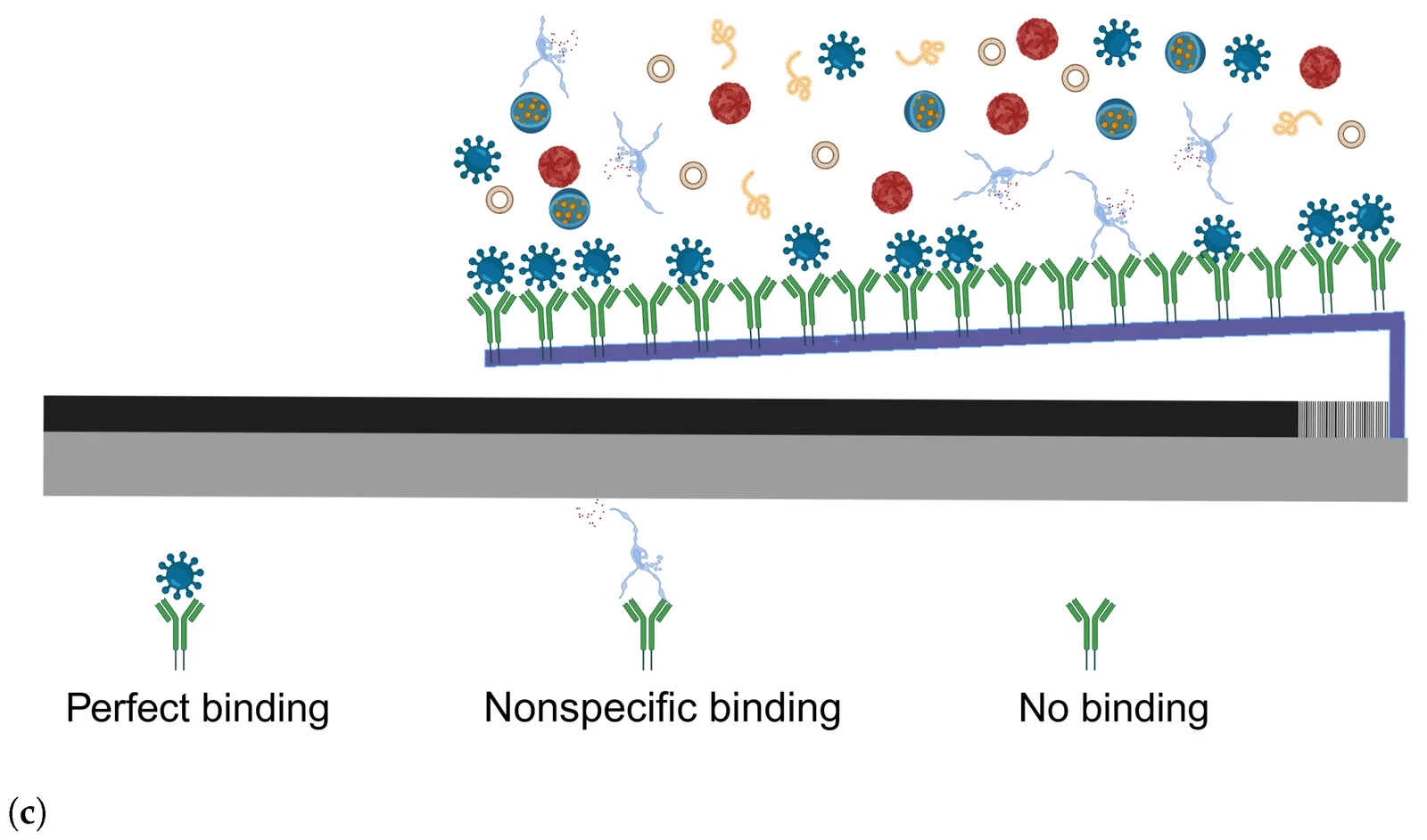
(**a**) Three-dimensional architecture of the BioMOEMS Platform, (**b**) schematic cross-sectional view of the functionalized sensing region in the presence of a biological sample, (**c**) illustration of biomolecular capture mechanisms and the resulting mechanical deflection of the cantilever.

To address these limitations, this paper presents a novel label-free BioMEMS platform based on an unbalanced DBR-terminated Michelson interferometer [30] on a silicon-on-insulator (SOI) platform, as illustrated in Figure 1. The proposed architecture introduces a highly sensitive optical transduction mechanism that converts biomolecular interactions into amplified spectral responses, enabling compact, fully integrated, and label-free biosensing. The proposed platform is applicable to a broad range of biomolecular detection tasks, including biomarker quantification and monitoring of biomolecular activation states. The remainder of the paper is organized as follows. In Section 2, we present the proposed structure and its working principles. A comparative study is then carried out in Section 3, followed by the conclusion.

## 2. BioMOEMS Structure and Operational Principles

The proposed integrated MOEMS architecture relies on a two-level transduction mechanism, in which mechanical and optical effects are sequentially coupled rather than independently optimized. The device integrates a flexible Parylene-C microcantilever with a Michelson interferometer, in which each arm consists of a single-pass waveguide terminated by a DBR mirror, forming a Michelson interferometer without internal Fabry–Perot cavities, to detect evanescent-field variations with high sensitivity (Figure 1a). This dual amplification mechanism generates an ultrasensitive spectral shift.

The first level relies on a highly flexible Parylene-C micro-cantilever, suspended at a nanometric distance above a waveguide. The capture of bioparticles induces a compressive surface stress [8] that bends the cantilever toward the waveguide (Figure 1) [29], strongly modulating the evanescent field.

The second level exploits a Michelson interferometric architecture integrated on a photonic chip, where the optical signal is split by a Multi-Mode Interference (MMI) coupler into a reference arm and a sensing arm, and both arms are terminated with a single DBR mirror, enabling Michelson interference without forming internal Fabry–Perot cavities. The perturbation of the evanescent field by the cantilever locally modifies the effective refractive index of the sensing waveguide, thereby inducing an intrinsic spectral shift in the corresponding cavity. The resulting phase difference between the arms generates a sensitive spectral shift in the Michelson interference fringes, enabling highly sensitive measurement of the cantilever deflection. The architecture and operating principle of the proposed device, including its functionalization with specific bioreceptors, are illustrated in Figure 1. The corresponding geometric dimensions and material properties are summarized in Table 1. Specifically, the waveguide dimensions (500 × 220 nm) were selected in strict accordance with standard commercial SOI foundry capabilities for single-mode TE propagation.

### 2.1. MEMS Design and Analysis

**Figure 2:**
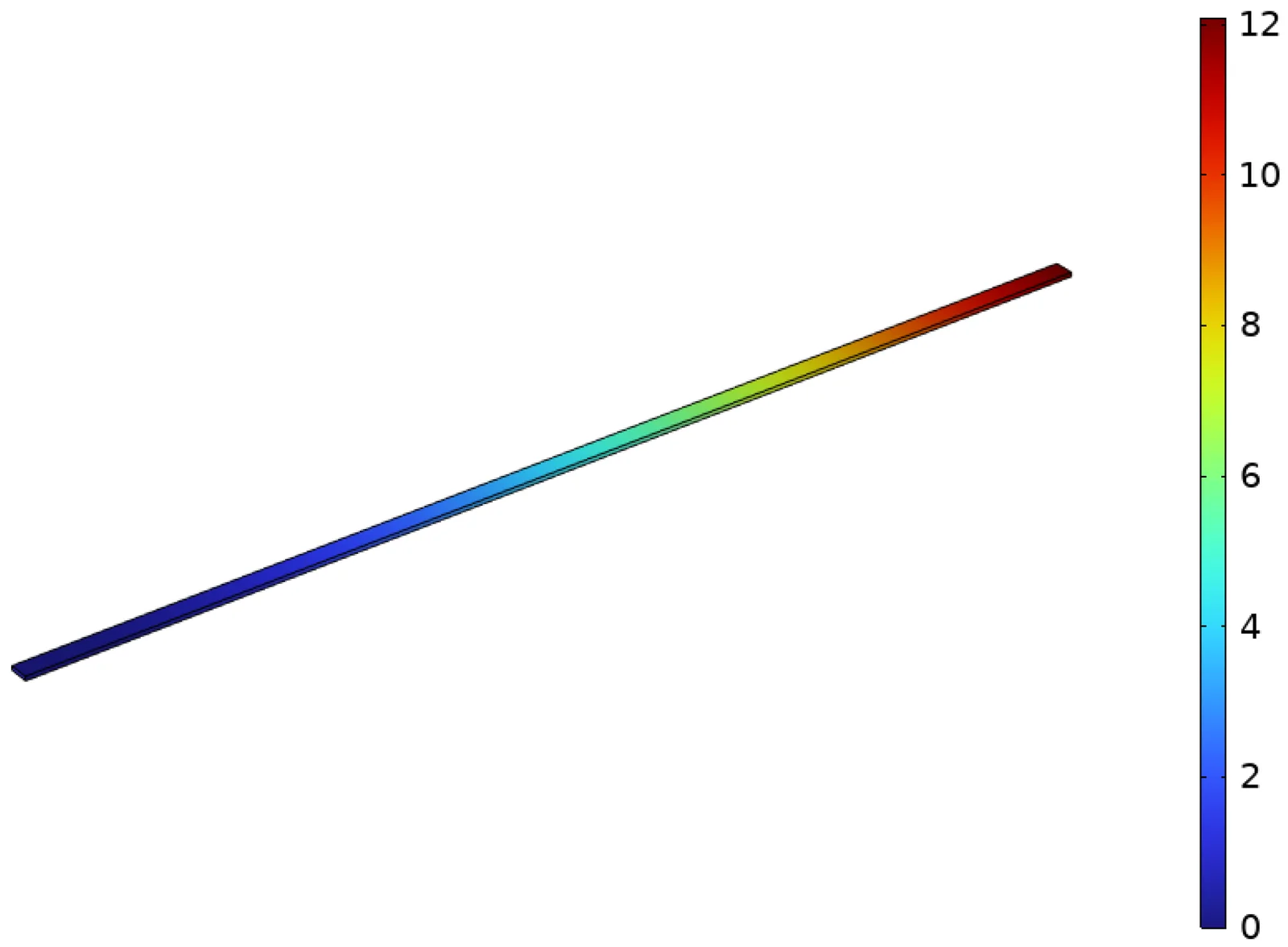
3D vertical displacement profile (µm) of the cantilever under compressive surface stress.

**Figure 3:**
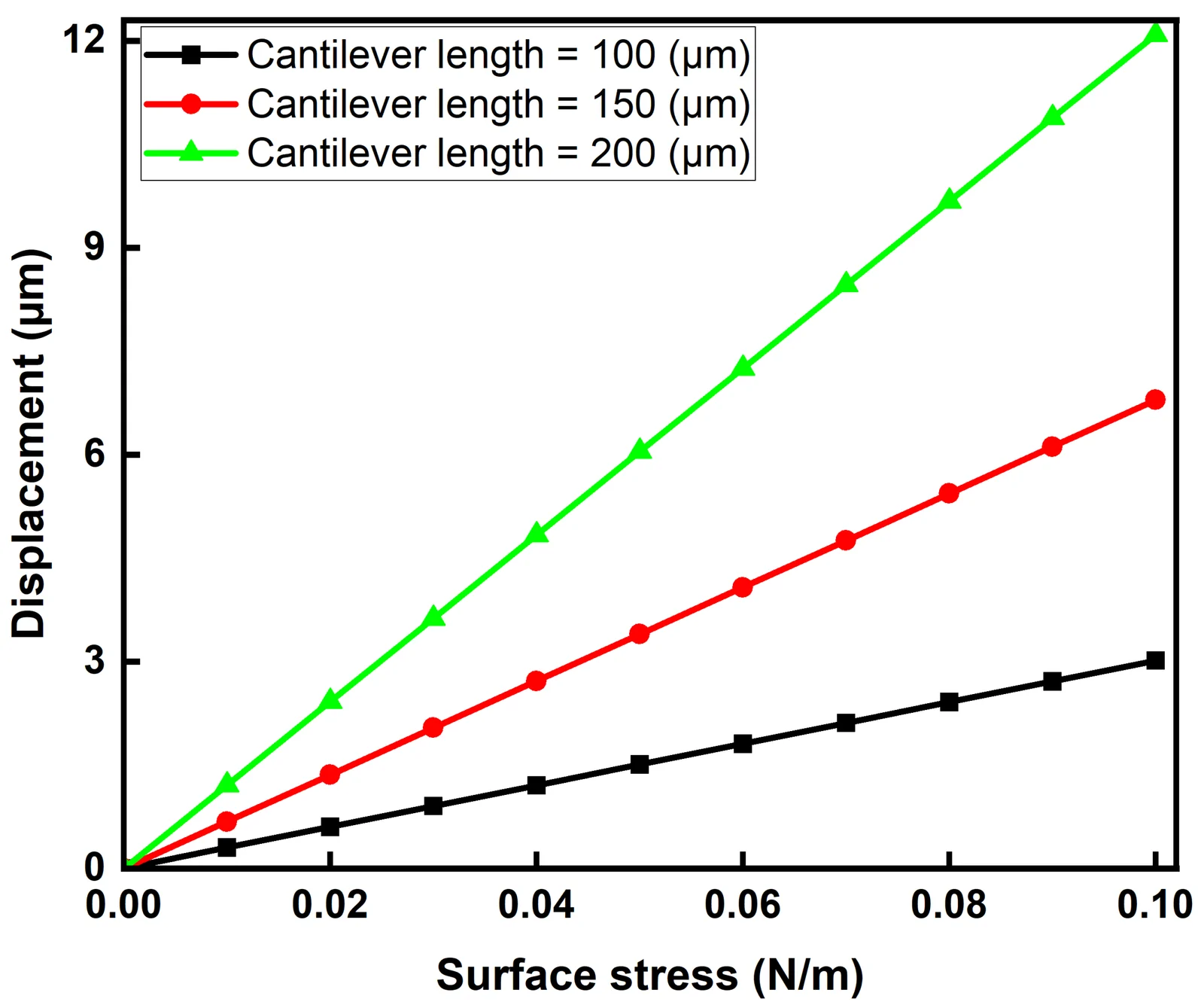
Maximum displacement as a function of applied surface stress for different lengths.

To design and analyze the MEMS component of the proposed architecture, a three-dimensional structural deformation analysis was performed using the COMSOL Multiphysics finite element simulation platform. The primary transducer was modeled as a clamped Parylene-C microcantilever, selected for its excellent biocompatibility and high mechanical flexibility. In the COMSOL model, biomolecule-induced stress was modeled as a uniform macroscopic boundary load on the top surface, statistically averaging out local non-uniformities of discrete binding [31,32]. The cantilever displacement as a function of the applied surface stress induced by biomolecular interactions was numerically evaluated for different cantilever lengths. Figure 2 illustrates the actual 3D vertical displacement profile obtained directly from the COMSOL simulations, visually confirming the structural bending behavior under the applied load. Concurrently, Figure 3 presents the maximum mechanical displacement as a function of the applied surface stress for varying cantilever lengths.

The mechanical sensitivity ($S_{m}$) is thus obtained by:

(1) $$S_{m}=\frac{\Delta z}{\Delta \sigma }$$

where Δz represents the vertical deflection and Δσ is the variation in surface stress. Hence, increasing the cantilever length enhances its mechanical sensitivity. Standard MEMS materials such as silicon (Si) and silicon nitride (Si_3_N_4_) possess relatively high Young’s moduli (∼160 GPa and ∼250 GPa, respectively), resulting in comparatively high structural stiffness and therefore limited deformation under a given surface stress. In contrast, Parylene-C exhibits a substantially lower Young’s modulus (4.5 GPa), providing greater mechanical compliance and, consequently, larger surface-stress-induced deflection for comparable cantilever geometries. Combined with its established biocompatibility and suitability for BioMEMS fabrication, this enhanced mechanical compliance makes Parylene-C particularly attractive for the proposed transduction mechanism. Furthermore, while a longer cantilever increases static sensitivity, it introduces severe physical limitations. Lengths exceeding 200 µm at sub-micron thicknesses are highly susceptible to stiction forces (such as capillary and Van der Waals forces) in liquid environments. Additionally, because the fundamental resonant frequency is inversely proportional to the square of the length ($f_{0}\propto 1/L^{2}$), excessive elongation drastically lowers $f_{0}$, rendering the device vulnerable to low-frequency environmental noise. Thus, 150 µm provides the optimal compromise between mechanical sensitivity, structural integrity, and dynamic stability. Based on this design, the mechanical sensitivity of the proposed biosensor is 67.935 µm/(N/m). Furthermore, the dynamic response of the BioMEMS sensor plays a crucial role in determining its operating bandwidth (BW). Accordingly, the fundamental resonant frequency ($f_{0}$) of the proposed device can be expressed as:

(2) $$f_{0}=\frac{1}{2\pi }\sqrt{\frac{k}{m_{\mathrm{eff}}}}$$

where *k* is the effective stiffness of the micro-cantilever and $m_{\mathrm{eff}}$ is the effective mass associated with the fundamental mode.

**Figure 4:**
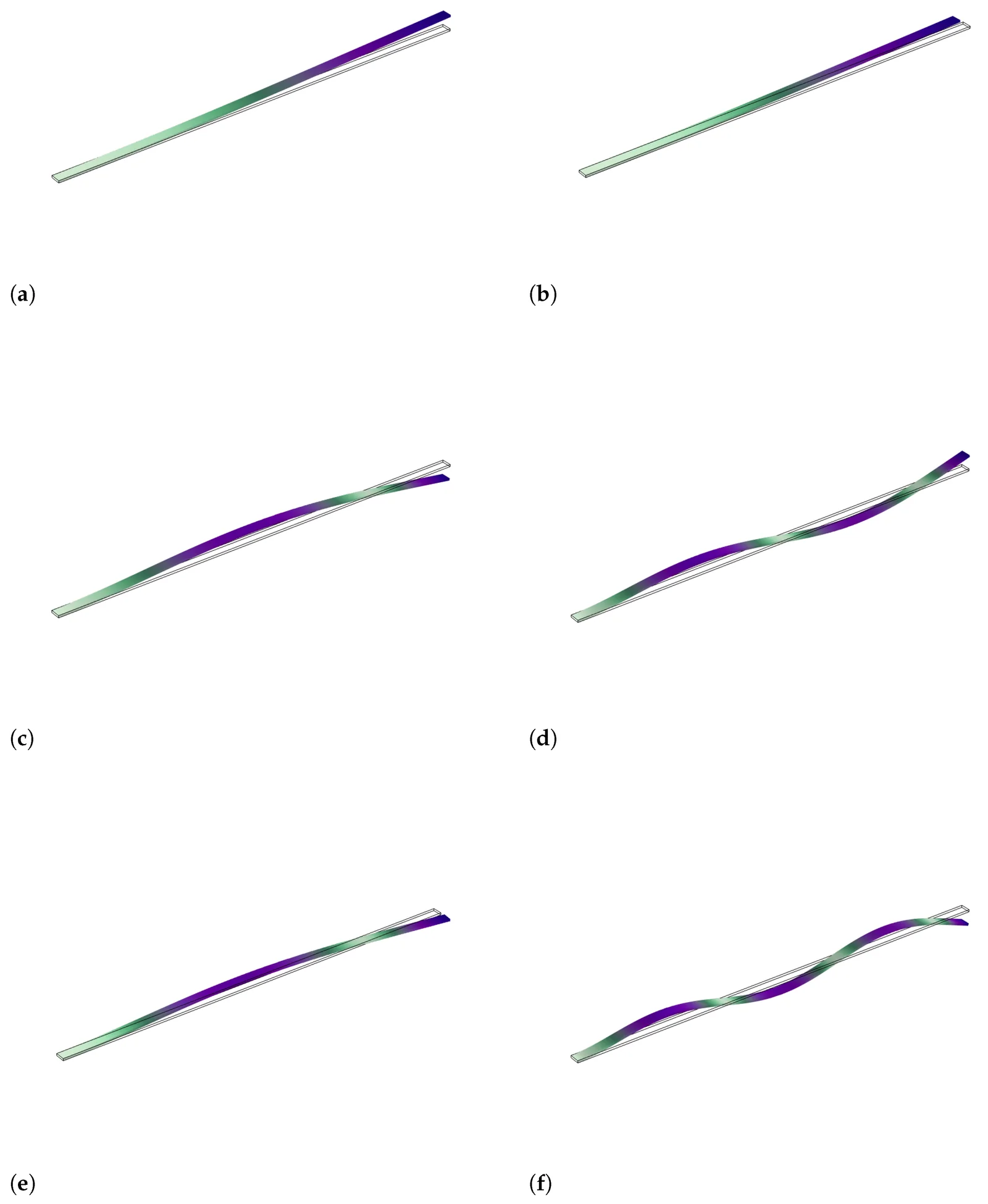
The first six resonance modes of the proposed BioMEMS: (**a**) $f_{1}$: 10.5 kHz, (**b**) $f_{2}$: 48.32 kHz, (**c**) $f_{3}$: 65.73 kHz, (**d**) $f_{4}$: 184.01 kHz, (**e**) $f_{5}$: 301.95 kHz and (**f**) $f_{6}$: 360.53 kHz.

**Figure 5:**
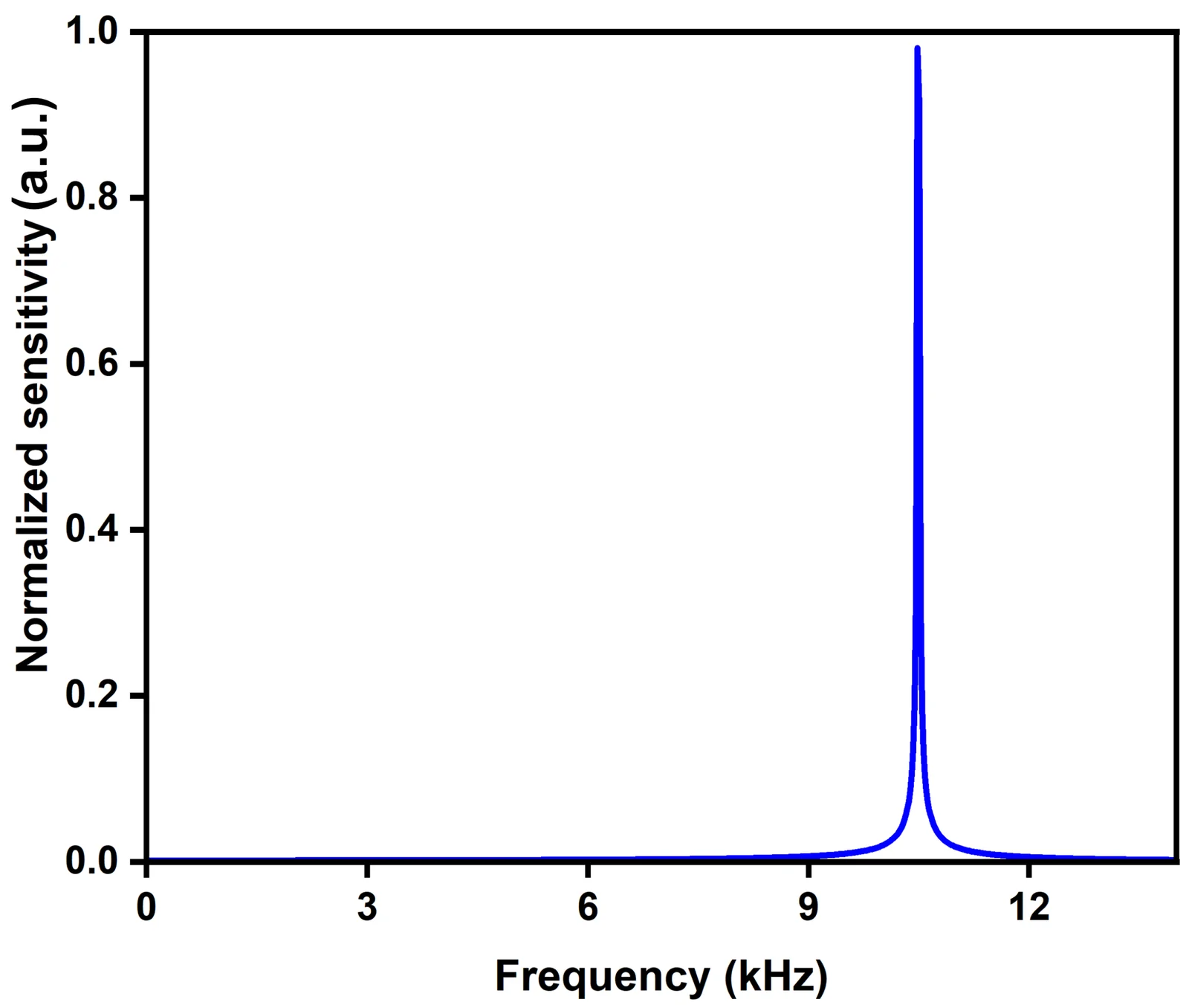
Frequency response of the device highlighting the first fundamental resonance mode.

The mechanical dynamic behavior of the proposed biosensor was investigated through modal and frequency response analyses. The first six resonant modes are presented in Figure 4, while the corresponding frequency response is shown in Figure 5. The numerical results are in good agreement with the analytical predictions, validating the proposed mechanical model. As observed in Figure 5, the frequency response remains essentially flat up to the vicinity of the first resonant mode, indicating a nearly constant mechanical sensitivity within this frequency range. Accordingly, the operating bandwidth of the proposed biosensor is estimated to be approximately 3 kHz.

The frequency-response analysis identifies the first mechanical resonance of the proposed cantilever at 10.5 kHz. The dynamic response of microcantilever-based sensors is inherently frequency dependent, with resonance-induced amplification becoming increasingly significant as the excitation frequency approaches the fundamental mechanical resonance [33]. Mouro et al. [33], for example, described the dynamic response of microcantilevers in terms of their resonance frequency, quality factor, and excitation frequency, showing the strong dependence of the vibration amplitude on the proximity to resonance. From an operational perspective, the mechanical resonance should therefore be distinguished from the lower-frequency region over which a comparatively stable response can be maintained. This distinction has also been adopted in MEMS frequency-response characterization; Ge and Cretu [34], for instance, separately characterized the mechanical resonant frequency and a lower-frequency 5% flat-response band, demonstrating that the useful operating range can be defined below the first mechanical resonance [34]. Based on this physical consideration and the simulated frequency response in Figure 5, an upper operating frequency of approximately 3 kHz was conservatively selected for the present design. This corresponds to $f/f_{0}\approx 0.286$, where $f_{0}=10.5$ kHz is the simulated fundamental resonant frequency, and therefore provides a substantial separation from the first resonance. The 3 kHz value is consequently considered a design-oriented upper operating-frequency limit intended to reduce resonance-induced dynamic effects, rather than an additional characteristic frequency or an experimentally determined bandwidth.

### 2.2. Optical Design and Analysis

**Figure 6:**
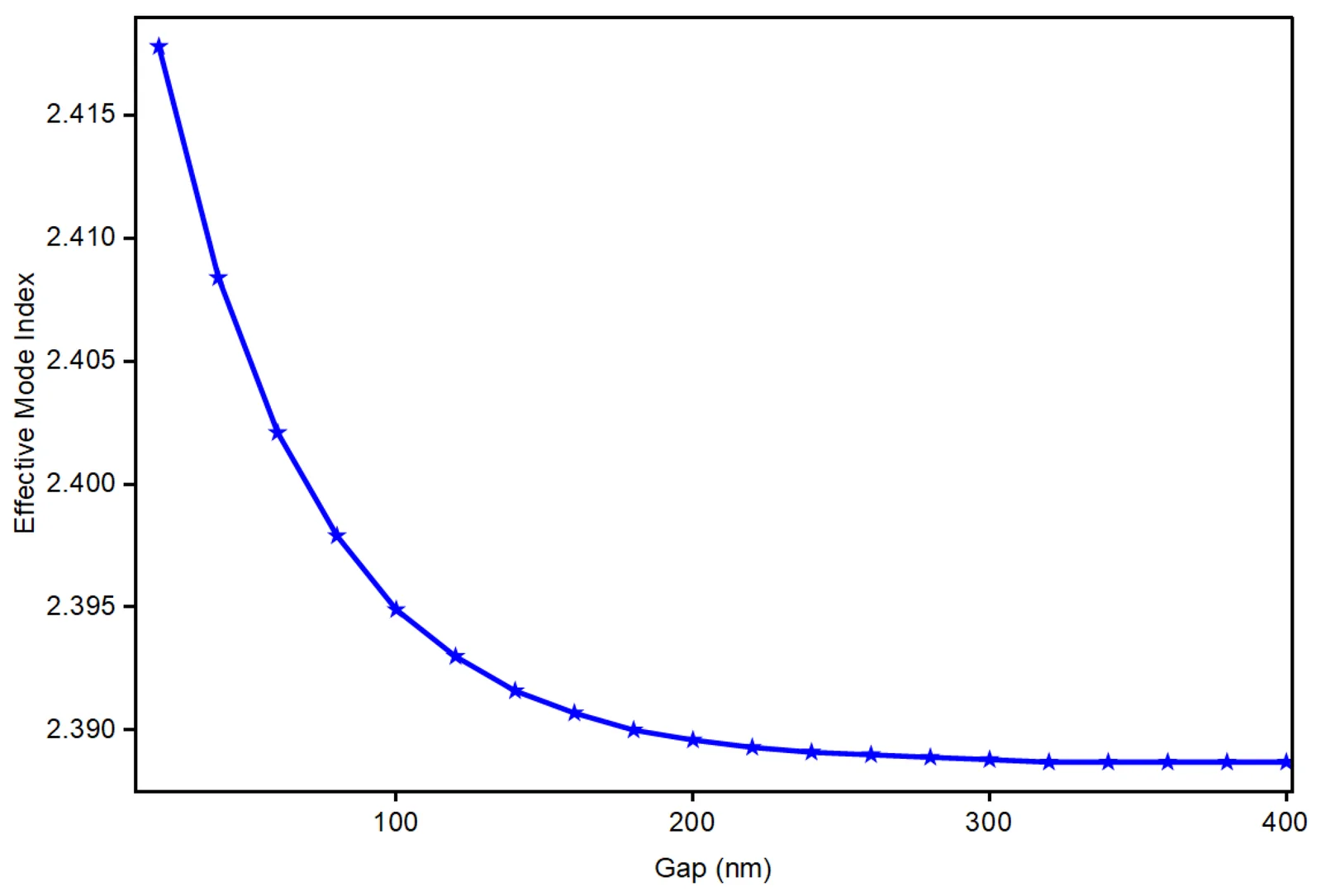
Evolution of the effective index of the guided mode as a function of the variation in the gap separating the cantilever from the waveguide.

To design and analyze the optical component of the proposed architecture, the optomechanical transduction of the cantilever deformation into an optical response was simulated using the Wave Optics module. The detection mechanism relies on the ability of mechanical motion to modify the propagation properties of light via evanescent coupling. To demonstrate this interaction, we conducted a 2D mode analysis to characterize the evolution of the effective index as a function of the gap variation (y-axis distance). As illustrated in Figure 6, the effective index decreases inherently exponentially as the gap increases, following the decay profile of the evanescent field in the cladding.

**Figure 7:**
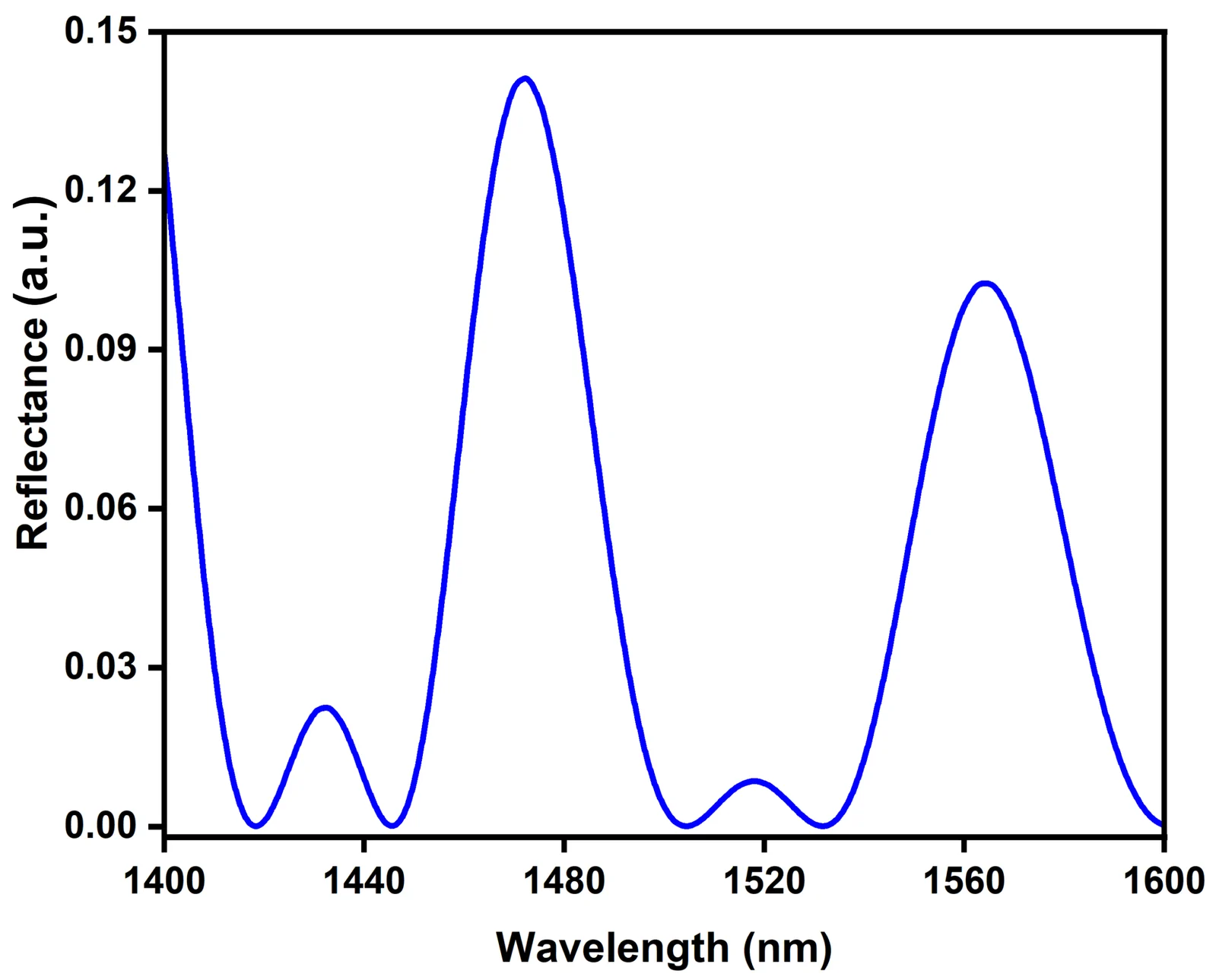
Calculated reflectance spectrum of the DBR-Michelson interferometer showing the dominant resonance peak and sidelobes.

**Figure 8:**
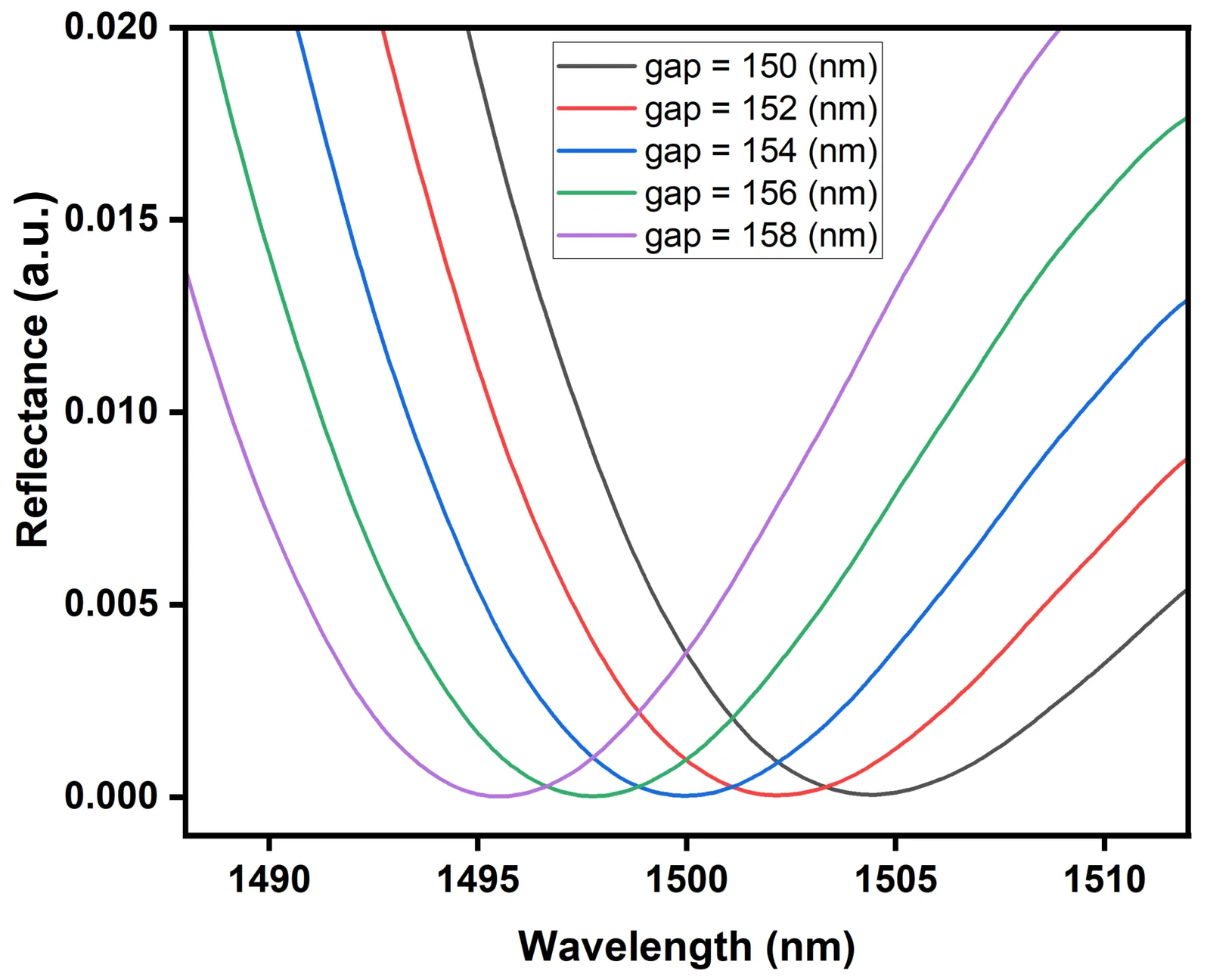
Spectral shift in the reflectance for different gap values.

From around 340 nm onward, the variation becomes nearly negligible, meaning the curve enters a saturation region where the effective index is practically constant. This curve allows for the definition of the transduction equation $n_{eff}=f\left(gap\right)$. This mathematical function and the physical detection limit are essential because they were directly injected into the 2D wave optics model to reliably convert a given mechanical displacement into a corresponding index variation on the sensing arm. The slight asymmetry between the two interferometer arms gives rise to the interference super-envelope bounded by the reflection stopband of the DBR mirrors, as illustrated in Figure 7 and Figure 8.

It is important to distinguish the initial fabrication gap from the gap range investigated in the optical sensing simulations. The initial gap of 250 nm corresponds to the nominal fabrication geometry and is defined by the sacrificial SiO_2_ layer used for cantilever release. In the optical analysis, reduced cantilever–waveguide gaps of 150–158 nm were parametrically investigated because this range provides appreciable evanescent-field interaction while exhibiting an approximately linear variation in the effective mode index over the considered interval. These discrete gap values therefore represent different mechanically displaced states of the cantilever and are used to evaluate their influence on the spectral response. To determine the spectral resolution of the device, the Full Width at Half Maximum (FWHM) was extracted from the raw reflectance spectrum. For the dominant resonance peak centered at 1472.2 nm, the half-maximum reflectance level was identified on the linear scale, and the FWHM was calculated as the wavelength difference ($\Delta \lambda =\lambda_{2}-\lambda_{1}$) between the two corresponding half-maximum crossing points, yielding a value of 27.6 nm. Furthermore, quality factor (*Q*) of the proposed device is given by:

(3) $$Q=\frac{\lambda_{r}}{\mathrm{FWHM}}$$

where $\lambda_{r}$ denotes the resonant wavelength of the optical mode, and FWHM represents the full width at half maximum of the resonance peak. In the proposed device, the resonant wavelength of the optical mode was calculated to be

$$\lambda_{r}=1472.2\;\mathrm{nm}$$

which yields:

$$Q=\frac{1472.2}{27.6}=53.34$$

Consequently, the specific binding of target biomolecules to the functionalized cantilever generates a surface stress that induces its mechanical deflection. This deformation reduces the gap between the cantilever and the underlying waveguide, thereby modifying the effective refractive index of the sensing arm through evanescent-field coupling. As a result, the resonance wavelength of the sensing cavity shifts, producing a corresponding spectral shift in the interference fringe minimum (Figure 8). The magnitude of the spectral shift is therefore related to the mechanically induced gap variation. Quantitative conversion of this spectral response into a target biomolecule concentration would require biomarker-specific calibration relating concentration and surface binding to the resulting surface stress, which is beyond the scope of the present study. Accordingly, the optical sensitivity of the proposed sensor is defined as:

(4) $$S_{o}=\frac{\Delta \lambda }{\Delta z}=1.115$$

where Δλ is the wavelength shift and Δz is the gap variation. The optical sensitivity was evaluated within the gap range of 150–158 nm, where the variation in the effective index in the sensing arm remains quasi-linear and exhibits only minimal fluctuations. The resonance wavelength obtained for each simulated gap value was fitted using a linear regression, and the slope of this fit was taken as the optical sensitivity $S_{o}$. This approach provides a robust estimation of the wavelength–gap conversion factor within the operating region. It is important to note that the high sensitivity of the proposed structure relies on the efficient phase-to-wavelength conversion of the Michelson interferometer. The small effective index variations induced by the cantilever deflection in the sensing arm are translated into measurable spectral shifts within the DBR reflection band, enabling ultra-low biomarker detection. Combining the mechanical and optical sensitivities, the overall sensitivity of the proposed BioMEMS sensor is given by:

$$S=S_{m}\times S_{o}=67.935\times 1.115=75.74\;µm/(N/m).$$

## 3. Comparative Study

To evaluate the performance of the proposed platform, its key characteristics are compared with those of representative state-of-the-art optical and optomechanical biosensors in Table 2. The proposed unbalanced Michelson interferometric architecture provides a narrow spectral response with a full width at half maximum (FWHM) of 27.6 nm and a quality factor of 53.34. In addition, the use of a flexible Parylene-C microcantilever enhances the mechanical response to surface-stress-induced deformation, resulting in a mechanical sensitivity of 67.935 µm/(N/m). By combining the mechanical amplification with interferometric optical interrogation, the proposed sensor achieves an overall sensitivity of 75.74 µm/(N/m). As summarized in Table 2, these results compare favorably with recently reported Fabry–Perot and MOEMS biosensors in terms of sensitivity while maintaining a compact integrated architecture.

While the proposed BioMEMS platform demonstrates promising performance through numerical analysis, experimental validation is required to fully assess its practical performance. The finite element model assumes ideal material properties and boundary conditions, whereas fabrication tolerances and residual stresses may lead to deviations from the predicted response. Furthermore, in realistic biomedical applications involving liquid media, viscous damping and added mass will lower the dynamic mechanical quality factor and bandwidth; however, because the sensing principle relies on steady-state static deflection, the static mechanical sensitivity remains unaffected. Future fabrication will utilize a 250 nm sacrificial ${\mathrm{SiO}}_{2}$ layer to ensure stiction-free release. Furthermore, different surface functionalization strategies (e.g., antibodies vs. aptamers) will yield varying baseline stresses and receptor layer thicknesses. Therefore, the generic differential surface stress (Δσ) utilized in our numerical model will require specific calibration based on the chosen experimental protocol to accurately assess true sensitivity and non-specific binding rates.

## 4. Conclusions

In conclusion, this work presents a novel label-free MOEMS biosensor employing an unbalanced DBR-Michelson interferometric architecture with a Parylene-C microcantilever. Numerical analyses demonstrate that the proposed dual-transduction architecture combines high mechanical compliance with enhanced optical interrogation, achieving an overall sensitivity of 75.74 µm/(N/m). These results demonstrate the potential of the proposed sensing approach for highly sensitive biomolecular detection. Future work will focus on the fabrication and in vitro experimental validation of the device under realistic biological conditions. The proposed architecture offers a promising platform for compact, integrated biosensors targeting disease diagnostics and point-of-care applications. It should be noted that in realistic liquid environments, fluidic damping will decrease the dynamic bandwidth, though the static mechanical sensitivity remains largely unaffected. Furthermore, environmental noise (thermomechanical, temperature drift) and non-specific binding cannot be intrinsically captured in this finite-element model and must be evaluated during future in vitro experimental calibrations, which are the subject of ongoing work.

## Figures and Tables

**Table 1:** Geometric parameters and physical properties of the model.

Component	Parameter	Symbol	Value
	Length	$L_{c}$	150 µm
	Width	$W_{c}$	3.6 µm
Cantilever	Thickness	$t_{c}$	0.78 µm
(Parylene-C)	Young’s Modulus	*E*	4.5 GPa
	Poisson’s Ratio	ν	0.4
	Density	ρ	1289 kg/m^3^
	Sensing arm length	$L_{\mathrm{sens}}$	150 µm
Waveguides	Reference arm length	$L_{\mathrm{ref}}$	145 µm
(Silicon)	Waveguide width	$W_{g}$	500 nm
	Silicon thickness	$t_{\mathrm{Si}}$	220 nm
DBR Mirrors	Period	Λ	273.57 nm
	Pairs	*N*	20
System/Optical	Initial gap	$g_{0}$	250 nm
	Silicon refractive index	$n_{\mathrm{Si}}$	3.48
	Silica refractive index	$n_{{\mathrm{SiO}}_{2}}$	1.44

**Table 2:** Comparison of the Functional Performances of the Proposed with Recent Contributions.

Ref.	Functional Mechanism	FWHM (nm)	Q	$S_{o}$	$S_{m}$ (µm/(N/m))	*S* (µm/(N/m))
[35]	All-optical Metamaterial	316	6.1	–	–	–
[36]	All-optical Plasmonic	53	23.77	–	–	–
[37]	MEMS Metamaterial	20	102.7	0.7	11.55	8.08
[27]	MEMS Photonic-crystal	0.8	1822.5	1.02	13.5	13.77
The proposed device	MEMS Interferometric	27.6	53.34	1.115	67.935	75.74

## Data Availability

The original contributions presented in this study are included in the article. Further inquiries can be directed to the corresponding authors.

## Abbreviations

The following abbreviations are used in this manuscript:

ELISA	Enzyme-Linked Immunosorbent Assay
PCR	Polymerase Chain Reaction
CDK	Cyclin-Dependent Kinase
MOEMS	Micro-Opto-Electro-Mechanical Systems
SPR	Surface Plasmon Resonance
SOI	Silicon-On-Insulator
MMI	Multimode Interference
DBR	Distributed Bragg Reflector
FWHM	Full Width at Half Maximum
Q	Quality Factor
FSR	Free Spectral Range
MEMS	Micro-Electro-Mechanical Systems
SLED	Superluminescent Light Emitting Diode
